# Food Insecurity and Micronutrient Deficiency in Adults: A Systematic Review and Meta-Analysis

**DOI:** 10.3390/nu15051074

**Published:** 2023-02-21

**Authors:** Sílvia Oliveira Lopes, Lívia Carvalho Sette Abrantes, Francilene Maria Azevedo, Núbia de Souza de Morais, Dayane de Castro Morais, Vivian Siqueira Santos Gonçalves, Edimar Aparecida Filomeno Fontes, Sylvia do Carmo Castro Franceschini, Silvia Eloiza Priore

**Affiliations:** Department of Nutrition and Health, Federal University of Viçosa, Viçosa 36570-900, MG, Brazil

**Keywords:** food security, nutritional status, micronutrients, systematic review, meta-analysis

## Abstract

Food insecurity is a public health problem as it affects a wide array of individuals in the population. It can be characterized by food deprivation, lack of essential nutrition, lack of dietary education, lack of adequate storage conditions, poor absorption, and poor overall nutrition. The relationship between food insecurity and micronutrient deficiency requires more effort to deepen and discuss the relationship. This systematic review aimed to evaluate the association between food insecurity and micronutrient deficiency in adults. The research was conducted according to PRISMA using the Medline/Pubmed, Lilacs/BVS, Embase, Web of Science, and Cinahl databases. Studies carried out with male and female adults were included, which investigated the correlation or association between food insecurity and the nutritional status of micronutrients. There were no publication year, country, or language restrictions. A total of 1148 articles were found, and 18 of these were included, carried out mainly on the American continent and with women. The most evaluated micronutrients were iron and vitamin A. Food insecurity was associated with nutrient deficiency in 89% (*n* = 16) of the studies. As a result of the meta-analysis, it was observed that there is a greater chance of anemia and low levels of ferritin among food insecure individuals. It is concluded that food insecurity is associated with micronutrient deficiency. Understanding these problems allows the creation of public policies capable of contributing to changes. Protocol registration: This review was registered on the PROSPERO-International Prospective Register of Systematic Reviews database—CRD42021257443.

## 1. Introduction

In its definition, the Food and Agriculture Organization of the United Nations (FAO) [1] establishes the relationship of four essential axes for the understanding of food security, namely availability, access, biological use, and food stability. The interrelation between these axes is mentioned in the definition provided by the World Food Summit [2], stating the importance of granting physical and economic access to quality food that meets the dietary needs and food preferences of everyone. Thus, food insecurity can be understood as when individuals and their families lack regular access to safe and nutritious food, which is necessary for their full development [3].

The 2030 Agenda for Sustainable Development recognizes the need to address food insecurity, hunger, and malnutrition worldwide. The positive projections of a decrease in this situation for the years 2009 to 2014/2015 failed to reach 2022. According to FAO, it is estimated that in 2021, 40.6% of the population (268 million people) faced moderate or severe food insecurity, an increase of 1.1% since 2020 [4].

Micronutrient deficiency may be related to the context of food (and nutritional) insecurity as another aggravating factor in the health status of insecure individuals who already have a poor self-perception of their health status [5]. Furthermore, this assessment should be considered in routine practices of diagnosis and health monitoring of individuals in situations of food insecurity, regardless of age group. It is known that this is not always true; therefore, this review aims to justify the importance of such measures. Food insecurity is influenced by issues, such as income, education, sex of the reference resident, and the presence of children in households, among other factors [6]. These issues associated with micronutrient deficiency contribute to the cyclic effect of food insecurity.

An example of the cyclic effect of food insecurity is the increase in the cost of food, which leads to difficulty in accessing as well as less diversity and quality, which are the basis for healthy eating. Insecure families tend to look for cheaper foods that generally offer greater satiety, including ultra-processed foods and products with greater energy density in their intake, to the detriment of fruits, vegetables, and meat/eggs, all which have micronutrient contents that favor good health [7]. The lack of access to a quality diet involving the aforementioned food groups can increase the chances of developing micronutrient deficiencies, contributing to an increase in overweight and cardiometabolic risk [8,9].

The relationship between food insecurity and micronutrient deficiency is complex since it requires an organization of measures, programs, and public policies that permeate food systems to contribute to dynamic processes from production to consumption. This is related to the cyclic effect of food insecurity and the global syndemic of obesity, malnutrition, and even climate change, which is defined as being “the synergisms between pandemics that coexist in time and space, interact with each other, and share common core social factors”. These food systems contribute both to obesity and malnutrition. In addition, the stimulus towards excessive consumption leads to increased emissions of greenhouse gases, which generate climate impacts that negatively affects the health conditions of the population [10].

Understanding the food system while considering the entire production chain that encompasses production, processing, distribution, and consumption, along with its relationship with the axes of food security, demonstrates the importance of intersectoral measures to address micronutrient deficiency [7].

Iron, iodine, zinc, folate, and vitamin A deficiencies are classified as “Generalized Global Micronutrient Deficiencies” [11]. These have a direct impact on bodily functions and can cause health problems, such as the reduction of the ability to learn, which can lead to a decrease in the individual’s productivity, making it difficult to access employment, among other factors, that can contribute to the cyclic effects of food insecurity [6,11]. Public measures aimed at improving access to natural foods of higher nutritional quality should be encouraged with the objective of promoting food security [12,13].

Faced with the issue of food insecurity and micronutrient deficiency and characterizing them as public health issues, the objective of this review was to evaluate the association between food insecurity and micronutrient deficiency in adults. Considering the above, this study aimed to provide a compilation justifying the importance of effective measures to address these two health assessment axes, in addition to the adult age group.

## 2. Methods

### 2.1. Study Design

This is a systematic review and meta-analysis study based on the recommendations of the PRISMA protocol, Preferred Reporting Items for Systematic Reviews and Meta-Analysis [14], registered in the International Prospective Register of Systematic Reviews (PROSPERO)—CRD42021257443.

The study question was developed based on the PECO acronym, where population (P) stands for adults, exposure (E) stands for food insecurity, comparison (C) stands for food security, and outcome (O) stands for food insecurity and micronutrient deficiency. Therefore, the present study was guided by the following question: “Is food insecurity associated with micronutrient deficiency in adult individuals?”

### 2.2. Eligibility

Studies that evaluated adults, regardless of gender, were included, in addition to studies that investigated the correlation or association between food insecurity and the nutritional status of micronutrients as assessed by biochemical tests. There were no restrictions on date, place, or language of publication.

Studies with qualitative analyses, reviews, and book chapters were excluded, as well as studies that evaluated pregnant and lactating women, specific conditions (genetic deficiencies in some micronutrient), or diseases, such as HIV and cancer. Studies that evaluated the nutritional status of micronutrients only by food consumption were excluded, as it is a subjective method of assessing the nutritional status.

### 2.3. Data Source and Search Strategy

The search strategy was developed and validated by the researchers based on the list of recommendations from the Peer Review of Electronic Search Strategies (PRESS) [15]. Subsequently, it was forwarded to an independent researcher for verification and suggestions (Supplementary Material File S1).

Five databases were used: Medline (via Pubmed), Lilacs, Embase, Web of Science, and Cinahl. The descriptors were localized using the controlled vocabulary from the DeCS and MeSH databases. Keywords were included from the indexed terms and their synonyms, and then, they were interleaved by the Boolean operators OR and AND for synonyms. The terms used were the following: “adult”, “young adult”, “middle aged”, “food insecurity”, “famine, iron”, “anemia”, iron-deficiency”, “vitamin B12 deficiency”, “vitamin A deficiency”, “growth disorders”, “iodine”, “malnutrition”, “incidence”, “cross-sectional studies” (Supplementary Material File S2). The search period was July 2022.

### 2.4. Selection of Studies

The selection was performed by two researchers (SOL and LCSA) independently using the Rayyan Software. Titles and abstracts were read in the first stage, and in cases of disagreement, a third researcher (NSM) was called to settle disagreements. In the second stage, the full text was read by two independent reviewers. In the absence of consensus, the third reviewer would once again be involved.

### 2.5. Data Extraction

Data extraction was carried out by three researchers (SOL, LCSA, and NSM), extracting author, year of publication, location, type of study, sample, assessment instruments and the prevalence of food insecurity, micronutrients assessed and/or assessment method, prevalence of deficiency, statistical tests used, adjustments, and the relationship between food insecurity and micronutrient deficiency. For those articles where the group studied (i.e., adults) was included in the total population, information regarding this group was requested from the researchers responsible for the article.

### 2.6. The Assessment of the Methodological Quality of the Studies Selected for Systematic Review

The instrument used to assess the risks of bias was the Joanna Briggs Institute’s recommendation tool for cross-sectional studies [16]. It is composed of the following eight questions: “Inclusion criteria clearly defined in the sample”; “Study subjects and environment described in detail”; “Exposure measured validly and reliably”; “Clearly defined objectives and inclusion and exclusion criteria”; “Confounders identified”; “Strategies for dealing with confounders”; “Validly and reliably measured results”; “Adequate statistical analysis” [16]. This assessment was not used as an inclusion criterion for the studies (Supplementary Material File S3).

### 2.7. Data Synthesis and Analysis

The measures of association between food insecurity and micronutrient deficiency were obtained from each study and were then pooled. The data were loaded into an Excel spreadsheet and then exported to the R Studio software, version 4.2.0, to perform the meta-analysis. The summarized measure was the odds ratio (OR), using the metabin function of the meta package. To assess publication bias, the funnel symmetry test was applied, performed by the funnel function [17]. The heterogeneity of studies included in each meta-analysis was assessed according to the square of the inverse variance (*I*^2^). For all analyses, the fixed effect was considered, taking into acccount the low heterogeneity observed (*I*^2^ < 25%). All results are summarized in the forest plot, using the forest function of the metafor package [18].

## 3. Results

**Figure 1:**
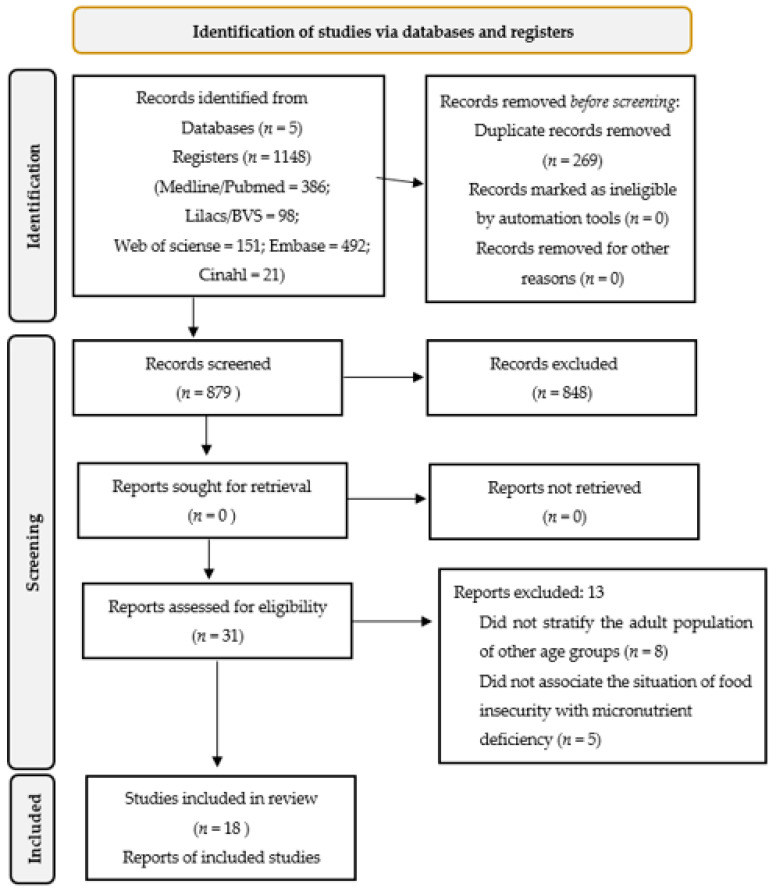
Scheme of the methodology adopted for the systematic review [14].

After following all the steps of the systematic review, 18 articles were selected (Figure 1). The studies selected for this review were published between 2001 and 2022, all with a cross-sectional design, carried out in different countries, with 56% (*n* = 10) [19,20,21,22,23,24,25,26,27,28] of them in the American continent, 28% (*n* = 5) [29,30,31,32,33] in Asia, and 16% (*n* = 3) [34,35,36] in the African continent. Of the studies, 72% (*n* = 13) were performed with females, 6% (*n* = 1) with males [22], and the rest with both males and females. Table 1 presents the description of the included studies.

Most studies evaluated food security with validated scales, 56% (*n* = 10) with the US Household Food Security Scale; 11% (*n* = 2) with the Latin American and Caribbean Food Security Scale [23,26], 5% (*n* = 1) with the Mexican Food Security Scale [27], 5% (*n* = 1) with the Abbreviated Household Food Security Scale [33], and 5% (*n* = 1) with the Brazilian Food Insecurity Scale [28] (Table 2); the others used tools to assess the situation of insecurity, such as the NHANES Family Questionnaire [19], the Childhood Hunger Identification Project Scale [35], and a structured questionnaire [30].

The results of micronutrients (iron, zinc, copper, and vitamins A, B12 (cobalamin), D, B9-Folate, C, and E reported in the studies were obtained using biochemical tests; with iron being the most evaluated micronutrient in 94% (*n* = 17) of the studies, using different markers individually or in combination. Of these, 83% (*n* = 15) were evaluated by hemoglobin, 39% (*n* = 7) by ferritin, 11% (*n* = 2) by the transferrin receptor, and 6% (*n* = 1) by serum iron. Vitamin A was evaluated in 28% (*n* = 5) of the studies, and 39% (*n* = 7) evaluated more than one micronutrient (Table 2).

Food insecurity was directly associated with nutrient deficiency processes in 89% (*n* = 16) of the studies. Of the studies that evaluated more than one micronutrient (*n* = 9), only one found an association between food insecurity and iron and vitamin D deficiency [33]. Anemia was the most recurrent outcome among the studies that evaluated the nutritional status of iron, representing 61% (*n* = 11). Associations were maintained after adjusting for sociodemographic and economic characteristics. The detailed results of the studies are described in Table 2.

**Figure 2:**
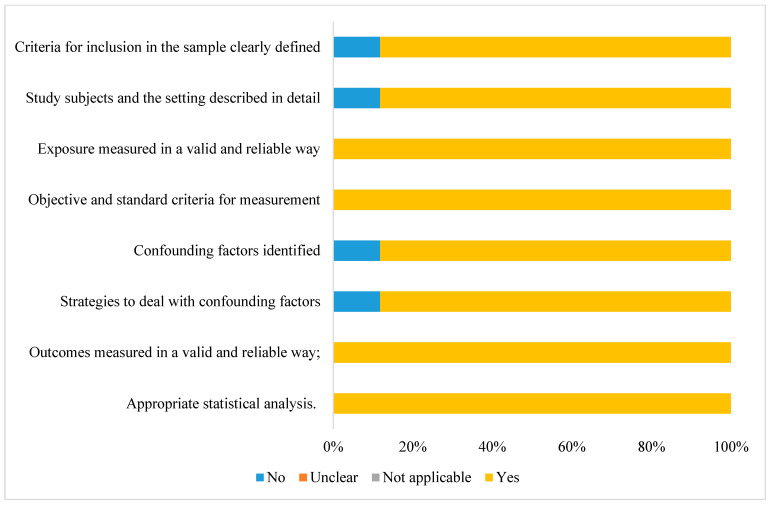
Risk of bias assessment for cross-sectional studies according to the Joanna Briggs Institute’s risk of bias assessment tool [16].

Regarding the risk of bias assessment, all studies had appropriate statistical analyses, the results were obtained reliably, there was valid measurement exposure, and they followed objective criteria (Figure 2). Among the studies, 78% (*n* = 14) met all the criteria evaluated, presenting a low risk of bias (Supplementary Material File S3).

**Figure 3:**
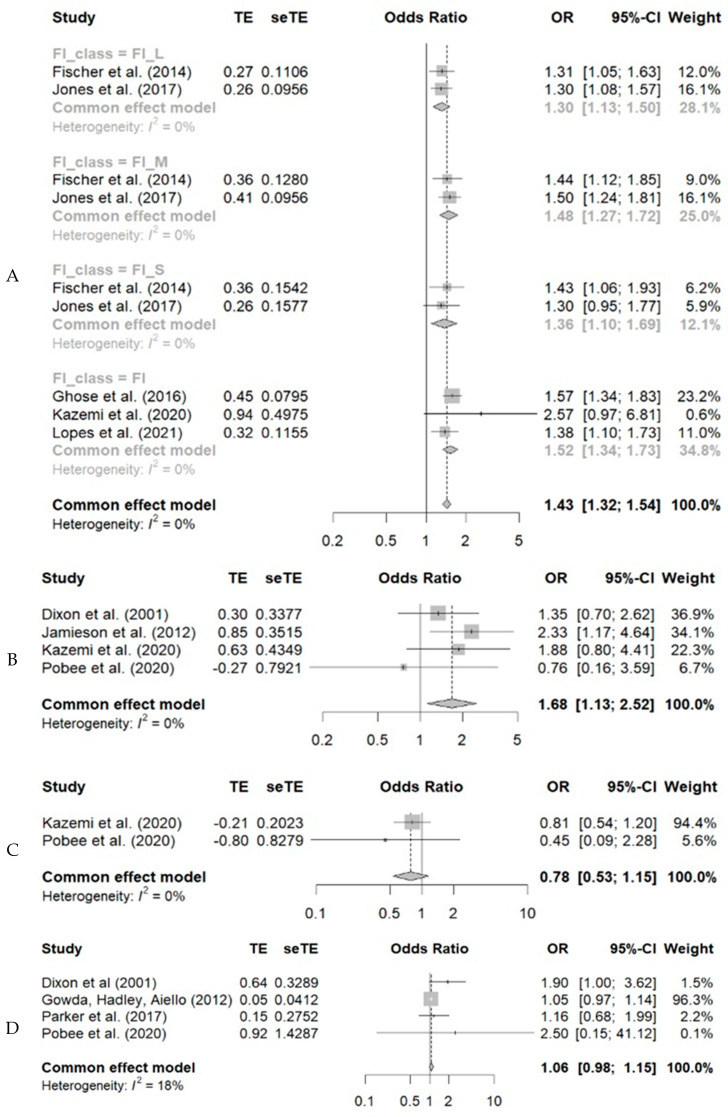
Meta-analysis of the association between food insecurity and anemia (**A**), low ferritin (**B**), vitamin D deficiency (**C**), and vitamin A (**D**) in adult individuals. The greater chance of anemia among individuals in a situation of food insecurity at any level of involvement. Thus, the chances of having anemia were 1.43 (1.34–1.73) higher among the insecure individuals. Individuals with food insecurity were 1.68 (1.13–2.52) times more likely to have low ferritin. For vitamins D and A, no statistical significance was observed [19,21,22,23,26,28,29,33,35,36].

In the systematization of the results, a greater chance of anemia was observed among individuals experiencing food insecurity at any level of involvement. Thus, considering the OR values (95% CI), the chances of having anemia were 1.43 (1.34–1.73) higher among insecure individuals. Likewise, food insecure individuals were 1.68 (1.13–2.52) times more likely to have low ferritin. For vitamins D and A, no statistical significance was observed (Figure 3).

## 4. Discussion

Most studies showed an association between food insecurity and micronutrient deficiency in adults, highlighting iron as the main mineral evaluated. Individuals in situations of food insecurity were more likely to be anemic and have low levels of ferritin, regardless of the level of insecurity they may have experienced. It should be noted that, in this review, the articles assessed the situation of food insecurity through the interviewee’s perception, using scales or basic questions. In addition to iron, the selected studies included the assessment of vitamins A, C, E, B12, and D and zinc and copper, demonstrating the importance of assessing other micronutrients as well, but the number of articles included in this review demonstrates the scarcity of publications that cover the issue of nutritional deficiencies through biochemical tests in adults and their relationship with food insecurity. For other micronutrients, whose deficiency is considered a public health problem, such as iodine, no studies were found that covered food insecurity.

Articles evaluating micronutrient deficiencies in children are more commonly found in the literature. The most evaluated adult population consists of females within reproductive age, as they are more vulnerable to deficiencies, which may have repercussions on pregnancy and consequently on child development [37]. Female vulnerability in the context of disabilities is reported by Darnton-Hill et al. [38] being related to menstrual losses, number of pregnancies, lower education, and greater poverty, which results in a higher risk of morbidity and mortality. For instance, anemia during pregnancy increases the risk of maternal mortality.

However, the need to assess the relationship between nutritional deficiencies and food insecurity in adults in general is emphasized as this age group is usually the first to be affected when there is food insecurity at home since they prioritize feeding children [39]. Still, they represent an important part of the population and are characterized by being economically active. Micronutrient deficiencies processes can lead to losses in the economy and in the health of the population in general.

Protein energy malnutrition and/or micronutrient deficiency (hidden hunger) leads to greater susceptibility to diseases. For females within reproductive age, it can impact fetal development, which according to Barker’s hypothesis, can result in the development of diseases in adulthood. The fetus exposed to low-nutrient availability can develop an adaptive response in the extrauterine environment that favors its development if the same nutrient supply condition is maintained. An insufficient or excessive supply of nutrients can predispose the child to diseases, such as obesity and others that affect metabolism, thus forming a cycle of diseases with food as an important determinant [40,41,42]. It is believed that factors related to the Becker hypothesis and its repercussions on the mother–child binomial may explain the higher prevalence of studies carried out with females in this review.

There is a challenge to be faced when linking micronutrient deficiency and food insecurity. In this context, inadequate nutrition or insufficient intake should be considered, related to issues of access, quality, and bioavailability of food, in addition to nutrient losses during preparation and/or consumption [43]. A study comparing families in situations of security and insecurity found lower energy consumption, protein, vitamins, and minerals among the insecure participants [44]. The findings of this review are reinforced by the study carried out to assess food insecurity in rural areas where the authors observed lower consumption of vegetables and fruit juice among the insecure, in addition to low-dietary diversity [45].

Dietary assessment is an indirect indicator of the nutritional status of micronutrients as well as the condition of food insecurity, being more prone to errors. However, according to Morais, Lopes, and Priore [6], food consumption indicators characterize the food insecurity proxy when food groups and/or nutrients do not meet dietary recommendations. Thus, they may be related to nutritional dystrophies and hidden hunger, characterized by anthropometric and biochemical assessments, allowing inferences to be made regarding the nutritional status of individuals. The use of direct indicators, such as biochemical tests to assess the concentrations of micronutrients, has less potential for error, and its singular or combined use with dietary assessment in population studies is interesting.

A micronutrient deficiency can also occur in combination with other deficiency processes. In a study carried out with members of the United States military service, the incidence of these disabilities was followed over the years, and there was an increase in rates over the years, especially among females and African Americans [46]. Another study carried out with schoolchildren found low urinary iodine excretion was common in the population with iron deficiency and anemia [47]. Therefore, the combined assessment of deficiency processes is important, considering food insecurity as one of the possible triggering factors.

Thus, the relationship between micronutrient deficiency and food insecurity is emphasized by the instability in the access and availability of quality food in sufficient quantity and in the biological use of nutrients by individuals, thus compromising the health of the adult population, as demonstrated by this review.

This review’s strengths include the inclusion of studies that used representative samples, which used validated instruments to assess food insecurity and micronutrients, in addition to the range of databases used to search for papers, the validation of the search strategy, as well as the performance of meta-analysis. Limitations of the present review was the studies did not consider the presence of parasites or other factors related to nutrient deficiency and were be cross-sectional as their design does not allow establishing a cause-and-effect relationship between food insecurity and micronutrient deficiencies.

## 5. Conclusions

An association between food insecurity and micronutrient deficiencies, especially iron and vitamin A, was found in the studies reviewed, where food insecure individuals are more likely to be anemic and have lower ferritin levels. These data are indicative of the need to explore the impact of food insecurity on micronutrient deficiencies, in addition to considering the relationship with other deficiencies that may coexist in individuals. This happens because deficiencies can cause social and economic costs to countries. Investing in population diagnoses and interventions to change this situation in all age groups can favor “breaking” the cycle of food insecurity.

## Figures and Tables

**Table 1:** Description of the included studies: author, year, country, sample, and objectives.

Author/Year	Country	Sample (Number, Sex, and Age)	Main Objective
Dixon et al. (2001) [19]	USA	6475, Males and females, 20–59 years	To examine whether dietary intake and serum nutrients differed between adults from food insufficient households (FIH) and adults from food sufficient households (FSH)
Egeland et al. (2011) [20]	Canada	2595, Males and females, ≥18 years	To assess biomarkers of nutritional status and nutrient intake from traditional foods (TF) and food security status among the Inuit in Canada
Gowda, Hadley, Aiello (2012) [21]	USA	12,191, Males and females, ≥18 years	To investigate whether food insecurity is associated with nutritional levels, inflammatory response, and altered immune function
Jamieson et al. (2012) [22]	Canada	994, Males, 18–39 years	To determine the prevalence of anemia, storage iron depletion, and iron overload, in addition to identifying correlates of iron status in Canadian Inuit males
Fischer et al. (2014) [23]	Mexico	11,205, Females, 21–49 years	To determine the association of household food insecurity with anemia in a nationally representative cross-sectional sample of Mexican females within reproductive age (12–49 years)
McDonald et al. (2015) [34]	Cambodia	Females, Mean age 29.6 ± 6.5 years	To assess household food insecurity and food diversity as correlates of maternal and child anthropometric status and anemia in rural Cambodia
Sekhar et al. (2016) [24]	USA	3617, Females, 22–49 years	To examine risk factors for iron deficiency anemia in a nationally representative sample of younger (12–21 years) and older (22–49 years) adult females.
Ghose et al. (2016) [29]	Bangladesh	5666, Females, 13–40 years	To investigate whether there is any association between household food insecurity and anemia among females within reproductive age in Bangladesh
Weigel et al. (2016) [25]	Ecuador	794, Females, <30 years (*n* = 344); 30–44 years (*n* = 327); ≥45 years (*n* = 123)	To investigate the association of household food insecurity with the nutritional status of adult females living in families with children in low-income neighborhoods in Quito, Ecuador
Parker et al. (2017) [35]	South Africa	1205, Females, 16–35 years	To determine the current vitamin A status of a nationally representative sample of females, comparing them with previous national data and determining the impact of sociodemographic aspects, diet, and body size on vitamin A status
Soofi et al. (2017) [30]	Pakistan	11,751, Females, 15–49 years	To determine the prevalence and possible factors associated with anemia, vitamin B12, and folate deficiencies in females within reproductive age
Jones et al. (2017) [26]	Mexico	10,760, Females, 20–49 years	To determine the association between household food insecurity and the co-occurrence of becoming overweight and having anemia among females within reproductive age in the Mexican population
Habib et al. (2018) [31]	Pakistan	7491, Females, 15–49 years	To investigate iron deficiency anemia in Pakistani females
Mastiholi et al. (2018) [32]	India	770, Females, 15–39 years	To assess food insecurity and the nutritional status of preconception females in a rural population in northern Karnataka
Murillo-Castillo et al. (2018) [27]	Mexico	116, Females, Mean age 36.4 ± 8.9 years	To determine whether food insecurity is associated with dietary and biochemical measures in mothers from northwestern Mexico, who depend mostly on fishing for their subsistence
Kazemi et al. (2020) [33]	Iran	266, Females, The mean age was 40.93 ± 11.1 years	To investigate the association between household food insecurity and anemia, iron deficiency, and vitamin D deficiency among females within reproductive age in East Azerbaijan, Iran
Pobee et al. (2020) [36]	Ghana	95, Females, 18–35 years	To examine the association between food insecurity and micronutrient status among Ghanaian females who are planning to become pregnant
Lopes et al. (2022) * [28]	Brazil	198, Males and females, 20–59 years	To determine the prevalence of anemia and associated factors in adults and elderly residents of the rural area of a city in Zona da Mata, Minas Gerais of Brazil

Fe = iron; Hb = hemoglobin; TF = traditional foods, * Data provided by the authors when decoupling the adult group from the total population.

**Table 2:** The instruments used to assess food insecurity, micronutrient deficiencies, and the prevalence across studies.

Author/Year	Evaluation		Association between FI and Micronutrient Deficiency **
	FI	Deficiency, Marker or Micronutrient	
Dixon et al. (2001) [19]	NHANES family questionnaire *	Ferritin	Young adults experiencing FI had lower serum concentrations of vitamin A and carotenoids than those experiencing FS; furthermore, elderly participants experiencing FI had lower concentrations of vitamin A and vitamin E than those in FS.
		Folate	
		Vitamin A, C, E, B12, D	
Egeland et al. (2011) [20]	Household Food Safety Survey—USDA	Hemoglobin	Female and male adults experiencing FI had lower serum ferritin concentrations; postmenopausal females who did not consume traditional foods had lower mean ferritin. Premenopausal females and males experiencing FI had lower hemoglobin values.
		Ferritin	
		Vitamin D	
Gowda, Hadley, Aiello, (2012) [21]	Household Food Safety Survey—USDA	Folate	NA
		Vitamin A, B12	
Jamieson et al. (2012) [22]	Household Food Safety Survey—USDA	Hemoglobin	FI was negatively associated with serum ferritin, and in insecure males, there was an increased risk of them having low or depleted iron stores.
		Ferritin	
		Transferrin receptor	
Fischer et al. (2014) [23]	Latin America and the Caribbean Food Security Scale	Hemoglobin	Females experiencing mild, moderate, and severe FI had a higher chance of being anemic.
McDonald et al. (2015) [34]	Household Food Safety Survey—USDA	Hemoglobin	FI was associated with anemia.
Sekhar et al. (2016) [24]	Household Food Safety Survey—USDA	Hemoglobin	FI was associated with anemia and predictors of iron deficiency.
		Ferritin	
		Transferrin receptor	
Ghose et al. (2016) [29]	Household Food Safety Survey—USDA	Hemoglobin	FI in females was associated with anemia.
Weigel et al. (2016) [25]	Household Food Safety Survey—USDA	Hemoglobin	FI was associated with anemia.
Parker et al. (2017) [35]	Scale of the Childhood Hunger Identification Project—South Africa	Vitamin A	Females experiencing FI were at an increased risk of Vitamin A deficiency.
Soofi et al. (2017) [30]	Structured Questionnaire—Pakistan	Hemoglobin	Females experiencing moderate FI were more likely to be anemic.
		Folate	
		Vitamin B12	
Jones et al. (2017) [26]	Latin America and the Caribbean Food Security Scale	Hermoglobin	FI was associated with anemia.
Habib et al. (2018) [31]	Household Food Safety Survey—USDA	Hemoglobin	FI in females was associated with iron-deficiency anemia.
		Ferritin	
		Vitamin A	
		Zinc	
Mastiholi et al. (2018) [32]	Household Food Safety Survey—USDA	Hemoglobin	FI was associated with anemia.
Murillo-Castillo et al. (2018) [27]	Mexican Food Security Scale	Hemoglobin	NA
Kazemi et al. (2020) [33]	Abbreviated Household Food Security Scale- Iran	Hemoglobin	FI was associated with anemia, iron deficiency, and vitamin D deficiency.
		Ferritin	
		Vitamin D	
Pobee et al. (2020) [36]	Household Food Safety Survey—USDA	Hemoglobin	FI was negatively associated with Vitamin A concentrations.
		Ferritin	
		Serum iron	
		Zinc	
		Copper	
		Vitamin A, D	
Lopes et al. (2022) [28]	Brazilian Scale of Food Insecurity	Hemoglobin	FI was associated with anemia.

FI = food insecurity; FS = food security; NA = no association; * seven specific questions about frequency and reasons for not eating; ** supplementary material with prevalence information.

## Supplementary Materials

The following supporting information can be downloaded at: https://www.mdpi.com/article/10.3390/nu15051074/s1, File S1: Evaluation with the Peer Review tool of Electronic Search Strategies (PRESS), File S2: Search strategy and article location, File S3: Risk of bias assessment.
