# Supplementary material for: Food Insecurity and Micronutrient Deficiency in Adults: A Systematic Review and Meta-Analysis
Source: Nutrients. 2023 Feb 21;15(5):1074. doi: 10.3390/nu15051074 (PMC10005365; doi:10.3390/nu15051074)
Supplement: Supplementary file 1 [file nutrients-15-01074-s001.zip › nutrients-2170591-supplementary.pdf]

Supplementary Material File S1. Evaluation with the Peer Review tool of Electronic Search Strategies (PRESS)

Peer Review of Electronic Search Strategies

PRESS Guideline — Search Submission & Peer Review Assessment SEARCH

SUBMISSION: THIS SECTION TO BE FILLED IN BY THE SEARCHER

Searcher: Lopes et al.	Email: silvia.lopes.nut@hotmail.com
Date submitted: 19.08.2021	Date requested by: 24.08.2021 [Maximum = 5 working days]

Atualização da busca 15 de julho de 2022

Systematic Review Title:

Food insecurity and the nutritional status of micronutrients in population studies with adults: systematic review and meta-analysis

This search strategy is…

	My PRIMARY (core) database strategy — First time submitting a strategy forsearch question and database
X	My PRIMARY (core) strategy — Follow-up review NOT the first time submitting astrategy for search question and database. If this is a response to peer review, itemize the changes made to the review suggestions
	SECONDARY search strategy— First time submitting a strategy for search questionand database
	SECONDARY search strategy — NOT the first time submitting a strategy for search question and database. If this is a response to peer review, itemize thechanges made to the review suggestions

Database

(i.e., MEDLINE, CINAHL…): *[mandatory]*

MEDLINE

Interface

(i.e., Ovid, EBSCO…): *[mandatory]*

PUBMED

Research Question

(Describe the purpose of the search) *[mandatory]*

Há relação entre a situação de segurança alimentar e deficiência de micronutrientes?

PICO Format

(Outline the PICOs for your question — i.e., Patient, Intervention, Comparison, Outcome, and Study Design — as applicable)

**P**	Adults
**E**	Food insecurity
**C**	Food security
**O**	Micronutrient deficiency
**S**	Cross-sectional studies

Inclusion Criteria

(List criteria such as age groups, study designs, etc., to be included) *[optional]*

Study with adults (20 - 59 years);

Studies that investigated the relationship or association between food insecurity and micronutrient deficiency;

Studies conducted in any date, language or location

Exclusion Criteria

(List criteria such as study designs, date limits, etc., to be excluded) *[optional]*

- Review studies, letters to the editors, qualitative analyses, case studies or book chapters;
- Studies with adults with any comorbidity that requires an increase or reduction in the metabolic demand of some analyzed nutrient, i.e. pregnant women, specific diseases such as HIV, Tuberculosis and cancer
- Articles that evaluated the nutritional status from food consumption by these methods being an estimate of the nutritional status of the micronutrient.

Was a search filter applied?

Yes x No □

If YES, which one(s) (e.g., Cochrane RCT filter, PubMed Clinical Queries filter)? Provide the source if this is a published filter. *[mandatory if YES to previous question — textbox]*

Cross-sectional studies

Other notes or comments you feel would be useful for the peer reviewer? ***[optional]***

No

Please copy and paste your search strategy here, exactly as run, including the number ofhits per line. ***[mandatory]***

(Add more space, as necessary.)

	DECS e sinônimos	LINHAS DA ESTRATÉGIA	NÚMERO DE ESTUDOS LOCALIZADOS
**P**	Adult<br>Adults<br>Young Adult<br>Adult, Young<br>Adults, Young<br>Young Adults	(((((Adult) OR (Adults)) OR (Young Adult)) OR (Adult, Young)) OR (Adults, Young)) OR (Young Adults)	8,228,803 results
	**AND**		
**E**	Food Insecurity<br>Food Insecurities<br>Insecurities, Food<br>Insecurity, Food	(((Food Insecurity) OR (Food Insecurities)) OR (Insecurities, Food)) OR (Insecurity, Food)	5,547 results
**C**	Não é necessário incluir descritores.	Não tem	
	**AND**		
**O**	Famine, Occult<br>Iron<br>Iron-56<br>Iron 56<br>Iron Deficiency<br>Anemia, Iron-Deficiency<br>Anemia, Iron Deficiency<br>Iron-Deficiency Anemia<br>Iron Deficiency Anemia<br>Iron-Deficiency Anemias<br>Iron Deficiency Anemias<br>Anemias, Iron-Deficiency<br>Anemias, Iron Deficiency<br>Vitamin B 12 Deficiency<br>Deficiencies, Vitamin B12<br>Deficiency, Vitamin B 12<br>Deficiency, Vitamin B12<br>Vitamin B12 Deficiencies<br>Vitamin B12 Deficiency<br>Vitamin A Deficiency<br>Deficiencies, Vitamin A<br>Deficiency, Vitamin A<br>Vitamin A Deficiencies	((Famine, Occult) OR (Iron)) OR (Iron-56)) OR (Iron 56)) OR (Iron Deficiency)) OR (Anemia, Iron-Deficiency)) OR (Anemia, Iron Deficiency)) OR (Iron-Deficiency Anemia)) OR (Iron Deficiency Anemia)) OR (Iron-Deficiency Anemias)) OR (Iron Deficiency Anemias)) OR (Anemias, Iron-Deficiency)) OR (Anemias, Iron Deficiency)) OR (Vitamin B 12 Deficiency)) OR (Deficiencies, Vitamin B12)) OR (Deficiency, Vitamin B 12)) OR (Deficiency, Vitamin B12)) OR (Vitamin B12 Deficiencies)) OR (Vitamin B12 Deficiency)) OR (Vitamin A Deficiency)) OR (Deficiencies, Vitamin A)) OR (Deficiency, Vitamin A)) OR (Vitamin A Deficiencies)) OR (Zinc Deficiency)) OR (Growth Disorders)) OR (Milk, Human/chemistry)) OR (Zinc/deficiency)) OR (Iodine Deficiency)) OR (Iodine Deficiency, Primary)) OR (Hypothyroidism,	521,820 results

	Zinc Deficiency<br>Growth Disorders<br>Milk, Human/chemistry<br>Zinc/deficiency<br>Iodine Deficiency<br>Iodine Deficiency, Primary<br>Hypothyroidism, Congenital<br>Cretinism<br>Endemic Cretinism<br>Cretinism, Endemic<br>Fetal Iodine Deficiency Disorder<br>Myxedema, Congenital<br>Malnutrition<br>Nutritional Deficiency<br>Nutritional Deficiencies<br>Undernutrition<br>Malnourishment<br>Malnourishments	Congenital)) OR (Cretinism)) OR (Endemic Cretinism)) OR (Cretinism, Endemic)) OR (Fetal Iodine Deficiency Disorder)) OR (Myxedema, Congenital)) OR (Malnutrition)) OR (Nutritional Deficiency)) OR (Nutritional Deficiencies)) OR (Undernutrition)) OR (Malnourishment)) OR (Malnourishments)	

*Utilize as adaptações do acrônimo conforme a necessidade.

BASE DE DADOS	ESTRATÉGIA 1	NÚMERO DE ESTUDOS LOCALIZADOS
PUBMED	(((((((Adult) OR (Adults)) OR (Young Adult)) OR (Adult, Young)) OR (Adults, Young)) OR (Young Adults)) AND ((((Food Insecurity) OR (Food Insecurities)) OR (Insecurities, Food)) OR (Insecurity, Food))) AND (((((((((((((((((((((((((((((((((((((((Famine, Occult) OR (Iron)) OR (Iron-56)) OR (Iron 56)) OR (Iron Deficiency)) OR (Anemia, Iron-Deficiency)) OR (Anemia, Iron Deficiency)) OR (Iron-Deficiency Anemia)) OR (Iron Deficiency Anemia)) OR (Iron-Deficiency Anemias)) OR (Iron Deficiency Anemias)) OR (Anemias, Iron-Deficiency)) OR (Anemias, Iron Deficiency)) OR (Vitamin B 12 Deficiency)) OR (Deficiencies, Vitamin B12)) OR (Deficiency, Vitamin B 12)) OR (Deficiency, Vitamin B12)) OR (Vitamin B12 Deficiencies)) OR (Vitamin B12 Deficiency)) OR (Vitamin A Deficiency)) OR (Deficiencies, Vitamin A)) OR (Deficiency, Vitamin A)) OR (Vitamin A Deficiencies)) OR (Zinc Deficiency)) OR (Growth Disorders)) OR (Milk, Human/chemistry)) OR (Zinc/deficiency)) OR (Iodine Deficiency)) OR (Iodine Deficiency, Primary)) OR (Hypothyroidism, Congenital)) OR (Cretinism)) OR (Endemic Cretinism)) OR (Cretinism, Endemic)) OR (Fetal Iodine Deficiency Disorder)) OR (Myxedema, Congenital)) OR (Malnutrition)) OR (Nutritional Deficiency)) OR (Nutritional Deficiencies)) OR (Undernutrition)) OR (Malnourishment)) OR (Malnourishments))	386 results

PEER REVIEW ASSESSMENT: THIS SECTION TO BE FILLED IN BY THE REVIEWER

**Reviewer: Ana Claudia Morais Godoy Figueiredo**	**Email: aninha_m_godoy@hotmail.com**		**Date completed: 19.08.2021**
**1. TRANSLATION**			
	A. No revisions	**X**	
	B. Revision(s) suggested	□	
	C. Revision(s) required	□	

If “B” or “C,” please provide an explanation or example:

**2. BOOLEAN AND PROXIMITY OPERATORS**			
	A. No revisions	**X**	
	B. Revision(s) suggested	□	
	C. Revision(s) required	□	

If “B” or “C,” please provide an explanation or example:

**3. SUBJECT HEADINGS**			
	A. No revisions	□	
	B. Revision(s) suggested	**X**	
	C. Revision(s) required	□	

If “B” or “C,” please provide an explanation or example:

Sugiro que sejam avaliados os seguintes termos:

1) Incluir o termo Middle Aged e termo similar (Middle Age) para população do PECOS uma vez que inclui adultos com idade inferior a 45 anos. Os estudos com idosos serão excluídos na fase da leitura de títulos e resumos;
2) Incluir o termo Famine para o outcome do PECOS.
3) Incluir termo Zinc para o outcome do PECOS.
4) Incluir termos MESH Similares de Growth Disorders:
   Disorder, Growth
   Growth Disorder
   Stunting
   Stuntings
   Stunted Growth
   Growth, Stunted
5) Sugiro retirar o termo Milk, Human/chemistry – não é um aplicável para busca.
6) Retirar os seguintes descritores que são voltados para população infantil:
   Hypothyroidism, Congenital
   Cretinism
   Endemic Cretinism
   Cretinism, Endemic
   Fetal Iodine Deficiency Disorder
   Myxedema, Congenital
7) Incluir o termo Iodine e similares (Iodine-127; Iodine 127).

4. TEXT WORD SEARCHING			
	A. No revisions	**X**	
	B. Revision(s) suggested	□	
	C. Revision(s) required	□	

If “B” or “C,” please provide an explanation or example:

5. SPELLING, SYNTAX, AND LINE NUMBERS			
	A. No revisions	**X**	
	B. Revision(s) suggested	□	
	C. Revision(s) required	□	

If “B” or “C,” please provide an explanation or example:

6. LIMITS AND FILTERS			
	A. No revisions	□	
	B. Revision(s) suggested	**X**	
	C. Revision(s) required	□	

If “B” or “C,” please provide an explanation or example:

Devido ao fato de ser selecionado apenas estudos do tipo transversal, sugiro incluir o seguinte filtro por tipo de estudo:

(((Incidence[MeSH Terms]) OR (Incidence[Title/Abstract])) OR (Incidences[Title/Abstract])) OR (Secondary Attack Rate[Title/Abstract])) OR (Attack Rate, Secondary[Title/Abstract])) OR (Rate, Secondary Attack[Title/Abstract])) OR (Secondary Attack Rates[Title/Abstract])) OR (Incidence Proportion[Title/Abstract])) OR (Incidence Proportions[Title/Abstract])) OR (Proportion, Incidence[Title/Abstract])) OR (Attack Rate[Title/Abstract])) OR (Attack Rates[Title/Abstract])) OR (Rate, Attack[Title/Abstract])) OR (Cumulative Incidence[Title/Abstract])) OR (Cumulative Incidences[Title/Abstract])) OR (Incidence, Cumulative[Title/Abstract])) OR (Incidence Rate[Title/Abstract])) OR (Incidence Rates[Title/Abstract])) OR (Rate, Incidence[Title/Abstract])) OR (Person-time Rate[Title/Abstract])) OR (Person time Rate[Title/Abstract])) OR (Person-time Rates[Title/Abstract])) OR (Rate, Person-time[Title/Abstract])) OR (Cross-Sectional Studies[MeSH Terms])) OR (Cross-Sectional Studies[Title/Abstract])) OR (Cross Sectional Studies[Title/Abstract])) OR (Cross-Sectional Study[Title/Abstract])) OR (Studies, Cross-Sectional[Title/Abstract])) OR (Study, Cross-Sectional[Title/Abstract])) OR (Cross Sectional Analysis[Title/Abstract])) OR (Analyses, Cross Sectional[Title/Abstract])) OR (Cross Sectional Analyses[Title/Abstract])) OR (Disease Frequency Surveys[Title/Abstract])) OR (Cross-Sectional Survey[Title/Abstract])) OR (Cross Sectional Survey[Title/Abstract])) OR (Cross-Sectional Surveys[Title/Abstract])) OR (Survey, Cross-Sectional[Title/Abstract])) OR (Surveys, Cross-Sectional[Title/Abstract])) OR (Surveys, Disease Frequency[Title/Abstract])) OR (Disease Frequency Survey[Title/Abstract])) OR (Survey, Disease Frequency[Title/Abstract])) OR (Analysis, Cross-Sectional[Title/Abstract])) OR (Analyses, Cross-Sectional[Title/Abstract])) OR (Analysis, Cross Sectional[Title/Abstract])) OR (Cross-Sectional Analyses[Title/Abstract])) OR (Cross-Sectional Analysis[Title/Abstract])) OR

(Prevalence Studies[Title/Abstract])) OR (Prevalence Study[Title/Abstract])) OR (Studies, Prevalence[Title/Abstract])) OR (Study, Prevalence[Title/Abstract])

Adicionalmente, recomendo utilizar o limite de busca para título e resumos.

**7. OVERALL EVALUATION (Note: If one or more "revision required" is noted above, the response below must be "revisions required".)**			
	A. No revisions	□	
	B. Revision(s) suggested	**X**	
	C. Revision(s) required	□	

Additional comments:

Verificar sugestões. Incluir no S do PECOS os descritores e filtro para estudo transversal.
Sugestão da estratégia de busca após as considerações – Total de 249 registros na base Pubmed em 19.08.2021:

Linha de busca	Registros
((((((((((Adult[MeSH Terms])) OR (Adult[Title/Abstract])) OR (Adults[Title/Abstract])) OR (Young Adult[Title/Abstract])) OR (Young Adult[MeSH Terms])) OR (Adult, Young[Title/Abstract])) OR (Adults, Young[Title/Abstract])) OR (Young Adults[Title/Abstract])) OR (Middle Aged[Title/Abstract])) OR (middle aged[MeSH Terms])) OR (Middle Age[Title/Abstract])	8,220,621
((((Food Insecurity[MeSH Terms]) OR (Food Insecurity[Title/Abstract])) OR (Food Insecurities[Title/Abstract])) OR (Insecurities, Food[Title/Abstract])) OR (Insecurity, Food[Title/Abstract])	5,830
(((famine[MeSH Terms]) OR (famine[Title/Abstract])) OR (Iron[MeSH Terms])) OR (iron[Title/Abstract])) OR (Iron-56[Title/Abstract])) OR (Iron 56[Title/Abstract])) OR (Iron Deficiency[Title/Abstract])) OR (Anemia, Iron-Deficiency[MeSH Terms])) OR (Anemia, Iron-Deficiency[Title/Abstract])) OR (Anemia, Iron Deficiency[Title/Abstract])) OR (Iron-Deficiency Anemia[Title/Abstract])) OR (Iron Deficiency Anemia[Title/Abstract])) OR (Iron-Deficiency Anemias[Title/Abstract])) OR (Iron Deficiency Anemias[Title/Abstract])) OR (Anemias, Iron-Deficiency[Title/Abstract])) OR (Anemias, Iron Deficiency[Title/Abstract])) OR (Vitamin B 12 Deficiency[MeSH Terms])) OR (Vitamin B 12 Deficiency[Title/Abstract])) OR (Deficiencies, Vitamin B12[Title/Abstract])) OR (Deficiency, Vitamin B 12[Title/Abstract])) OR (Deficiency, Vitamin B12[Title/Abstract])) OR (Vitamin B12 Deficiencies[Title/Abstract])) OR (Vitamin B12 Deficiency[Title/Abstract])) OR (Vitamin A Deficiency[MeSH Terms])) OR (Vitamin A Deficiency[Title/Abstract])) OR (Deficiencies, Vitamin A[Title/Abstract])) OR (Deficiency, Vitamin A[Title/Abstract])) OR (Vitamin A Deficiencies[Title/Abstract])) OR (Zinc Deficiency[Title/Abstract])) OR (Zinc[MeSH Terms])) OR (Zinc[Title/Abstract])) OR (Growth Disorders[MeSH Terms])) OR (Growth Disorders[Title/Abstract])) OR (Disorder, Growth[Title/Abstract])) OR (Growth Disorder[Title/Abstract])) OR (Stunting[Title/Abstract])) OR (Stuntings[Title/Abstract])) OR (Stunted Growth[Title/Abstract])) OR (Growth, Stunted[Title/Abstract])) OR (Iodine[MeSH Terms])) OR (Iodine[Title/Abstract])) OR (Iodine-127[Title/Abstract])) OR (Iodine 127[Title/Abstract])) OR (Iodine Deficiency[Title/Abstract])) OR (Iodine Deficiency, Primary[Title/Abstract])) OR (Malnutrition[MeSH Terms])) OR (Malnutrition[Title/Abstract])) OR (Nutritional	666,247

Deficiency[Title/Abstract])) OR (Nutritional Deficiencies[Title/Abstract])) OR (Undernutrition[Title/Abstract])) OR (Malnourishment[Title/Abstract])) OR (Malnourishments[Title/Abstract])	
(((Incidence[MeSH Terms]) OR (Incidence[Title/Abstract])) OR (Incidences[Title/Abstract])) OR (Secondary Attack Rate[Title/Abstract])) OR (Attack Rate, Secondary[Title/Abstract])) OR (Rate, Secondary Attack[Title/Abstract])) OR (Secondary Attack Rates[Title/Abstract])) OR (Incidence Proportion[Title/Abstract])) OR (Incidence Proportions[Title/Abstract])) OR (Proportion, Incidence[Title/Abstract])) OR (Attack Rate[Title/Abstract])) OR (Attack Rates[Title/Abstract])) OR (Rate, Attack[Title/Abstract])) OR (Cumulative Incidence[Title/Abstract])) OR (Cumulative Incidences[Title/Abstract])) OR (Incidence, Cumulative[Title/Abstract])) OR (Incidence Rate[Title/Abstract])) OR (Incidence Rates[Title/Abstract])) OR (Rate, Incidence[Title/Abstract])) OR (Person-time Rate[Title/Abstract])) OR (Person time Rate[Title/Abstract])) OR (Person-time Rates[Title/Abstract])) OR (Rate, Person-time[Title/Abstract])) OR (Cross-Sectional Studies[MeSH Terms])) OR (Cross-Sectional Studies[Title/Abstract])) OR (Cross Sectional Studies[Title/Abstract])) OR (Cross-Sectional Study[Title/Abstract])) OR (Studies, Cross-Sectional[Title/Abstract])) OR (Study, Cross-Sectional[Title/Abstract])) OR (Cross Sectional Analysis[Title/Abstract])) OR (Analyses, Cross Sectional[Title/Abstract])) OR (Cross Sectional Analyses[Title/Abstract])) OR (Disease Frequency Surveys[Title/Abstract])) OR (Cross-Sectional Survey[Title/Abstract])) OR (Cross Sectional Survey[Title/Abstract])) OR (Cross-Sectional Surveys[Title/Abstract])) OR (Survey, Cross-Sectional[Title/Abstract])) OR (Surveys, Cross-Sectional[Title/Abstract])) OR (Surveys, Disease Frequency[Title/Abstract])) OR (Disease Frequency Survey[Title/Abstract])) OR (Survey, Disease Frequency[Title/Abstract])) OR (Analysis, Cross-Sectional[Title/Abstract])) OR (Analyses, Cross-Sectional[Title/Abstract])) OR (Analysis, Cross Sectional[Title/Abstract])) OR (Cross-Sectional Analyses[Title/Abstract])) OR (Cross-Sectional Analysis[Title/Abstract])) OR (Prevalence Studies[Title/Abstract])) OR (Prevalence Study[Title/Abstract])) OR (Studies, Prevalence[Title/Abstract])) OR (Study, Prevalence[Title/Abstract])	1,373,564
SUGESTÃO DE ESTRATÉGIA DE BUSCA	
((((((((((((((Adult[MeSH Terms])) OR (Adult[Title/Abstract])) OR (Adults[Title/Abstract])) OR (Young Adult[Title/Abstract])) OR (Young Adult[MeSH Terms])) OR (Adult, Young[Title/Abstract])) OR (Adults, Young[Title/Abstract])) OR (Young Adults[Title/Abstract])) OR (Middle Aged[Title/Abstract])) OR (middle aged[MeSH Terms])) OR (Middle Age[Title/Abstract])) AND (((((Food Insecurity[MeSH Terms]) OR (Food Insecurity[Title/Abstract])) OR (Food Insecurities[Title/Abstract])) OR (Insecurities, Food[Title/Abstract])) OR (Insecurity, Food[Title/Abstract]))) AND (((famine[MeSH Terms]) OR (famine[Title/Abstract])) OR (Iron[MeSH Terms])) OR (iron[Title/Abstract])) OR (Iron-56[Title/Abstract])) OR (Iron 56[Title/Abstract])) OR (Iron Deficiency[Title/Abstract])) OR (Anemia, Iron-Deficiency[MeSH Terms])) OR (Anemia, Iron-Deficiency[Title/Abstract])) OR (Anemia, Iron Deficiency[Title/Abstract])) OR (Iron-Deficiency Anemia[Title/Abstract])) OR (Iron Deficiency Anemia[Title/Abstract])) OR (Iron-Deficiency Anemias[Title/Abstract])) OR (Iron Deficiency Anemias[Title/Abstract])) OR (Anemias, Iron-Deficiency[Title/Abstract])) OR (Anemias, Iron Deficiency[Title/Abstract])) OR (Vitamin B 12 Deficiency[MeSH Terms])) OR (Vitamin B 12 Deficiency[Title/Abstract])) OR (Deficiencies, Vitamin B12[Title/Abstract])) OR (Deficiency, Vitamin B 12[Title/Abstract])) OR (Deficiency, Vitamin B12[Title/Abstract])) OR (Vitamin B12 Deficiencies[Title/Abstract])) OR (Vitamin B12 Deficiency[Title/Abstract])) OR (Vitamin A Deficiency[MeSH Terms])) OR (Vitamin A Deficiency[Title/Abstract])) OR (Deficiencies, Vitamin	162

A[Title/Abstract])) OR (Deficiency, Vitamin A[Title/Abstract])) OR (Vitamin A Deficiencies[Title/Abstract])) OR (Zinc Deficiency[Title/Abstract])) OR (Zinc[MeSH Terms])) OR (Zinc[Title/Abstract])) OR (Growth Disorders[MeSH Terms])) OR (Growth Disorders[Title/Abstract])) OR (Disorder, Growth[Title/Abstract])) OR (Growth Disorder[Title/Abstract])) OR (Stunting[Title/Abstract])) OR (Stuntings[Title/Abstract])) OR (Stunted Growth[Title/Abstract])) OR (Growth, Stunted[Title/Abstract])) OR (Iodine[MeSH Terms])) OR (Iodine[Title/Abstract])) OR (Iodine-127[Title/Abstract])) OR (Iodine 127[Title/Abstract])) OR (Iodine Deficiency[Title/Abstract])) OR (Iodine Deficiency, Primary[Title/Abstract])) OR (Malnutrition[MeSH Terms])) OR (Malnutrition[Title/Abstract])) OR (Nutritional Deficiency[Title/Abstract])) OR (Nutritional Deficiencies[Title/Abstract])) OR (Undernutrition[Title/Abstract])) OR (Malnourishment[Title/Abstract])) OR (Malnourishments[Title/Abstract]))) AND ((Incidence[MeSH Terms]) OR (Incidence[Title/Abstract])) OR (Incidences[Title/Abstract])) OR (Secondary Attack Rate[Title/Abstract])) OR (Attack Rate, Secondary[Title/Abstract])) OR (Rate, Secondary Attack[Title/Abstract])) OR (Secondary Attack Rates[Title/Abstract])) OR (Incidence Proportion[Title/Abstract])) OR (Incidence Proportions[Title/Abstract])) OR (Proportion, Incidence[Title/Abstract])) OR (Attack Rate[Title/Abstract])) OR (Attack Rates[Title/Abstract])) OR (Rate, Attack[Title/Abstract])) OR (Cumulative Incidence[Title/Abstract])) OR (Cumulative Incidences[Title/Abstract])) OR (Incidence, Cumulative[Title/Abstract])) OR (Incidence Rate[Title/Abstract])) OR (Incidence Rates[Title/Abstract])) OR (Rate, Incidence[Title/Abstract])) OR (Person-time Rate[Title/Abstract])) OR (Person time Rate[Title/Abstract])) OR (Person-time Rates[Title/Abstract])) OR (Rate, Person-time[Title/Abstract])) OR (Cross-Sectional Studies[MeSH Terms])) OR (Cross-Sectional Studies[Title/Abstract])) OR (Cross Sectional Studies[Title/Abstract])) OR (Cross-Sectional Study[Title/Abstract])) OR (Studies, Cross-Sectional[Title/Abstract])) OR (Study, Cross-Sectional[Title/Abstract])) OR (Cross Sectional Analysis[Title/Abstract])) OR (Analyses, Cross Sectional[Title/Abstract])) OR (Cross Sectional Analyses[Title/Abstract])) OR (Disease Frequency Surveys[Title/Abstract])) OR (Cross-Sectional Survey[Title/Abstract])) OR (Cross Sectional Survey[Title/Abstract])) OR (Cross-Sectional Surveys[Title/Abstract])) OR (Survey, Cross-Sectional[Title/Abstract])) OR (Surveys, Cross-Sectional[Title/Abstract])) OR (Surveys, Disease Frequency[Title/Abstract])) OR (Disease Frequency Survey[Title/Abstract])) OR (Survey, Disease Frequency[Title/Abstract])) OR (Analysis, Cross-Sectional[Title/Abstract])) OR (Analyses, Cross-Sectional[Title/Abstract])) OR (Analysis, Cross Sectional[Title/Abstract])) OR (Cross-Sectional Analyses[Title/Abstract])) OR (Cross-Sectional Analysis[Title/Abstract])) OR (Prevalence Studies[Title/Abstract])) OR (Prevalence Study[Title/Abstract])) OR (Studies, Prevalence[Title/Abstract])) OR (Study, Prevalence[Title/Abstract]))

Supplementary Material File S2. Search strategy and article location

DATA BASE	STRATEGY	NUMBER OF LOCALIZED STUDIES
Medline	((((((((((((((Adult[MeSH Terms])) OR (Adult[Title/Abstract])) OR (Adults[Title/Abstract])) OR (Young Adult[Title/Abstract])) OR (Young Adult[MeSH Terms])) OR (Adult, Young[Title/Abstract])) OR (Adults, Young[Title/Abstract])) OR (Young Adults[Title/Abstract])) OR (Middle Aged[Title/Abstract])) OR (middle aged[MeSH Terms])) OR (Middle Age[Title/Abstract])) AND (((((Food Insecurity[MeSH Terms]) OR (Food Insecurity[Title/Abstract])) OR (Food Insecurities[Title/Abstract])) OR (Insecurities, Food[Title/Abstract])) OR (Insecurity, Food[Title/Abstract]))) AND ((famine[MeSH Terms]) OR (famine[Title/Abstract])) OR (Iron[MeSH Terms])) OR (iron[Title/Abstract])) OR (Iron-56[Title/Abstract])) OR (Iron 56[Title/Abstract])) OR (Iron Deficiency[Title/Abstract])) OR (Anemia, Iron-Deficiency[MeSH Terms])) OR (Anemia, Iron-Deficiency[Title/Abstract])) OR (Anemia, Iron Deficiency[Title/Abstract])) OR (Iron-Deficiency Anemia[Title/Abstract])) OR (Iron Deficiency Anemia[Title/Abstract])) OR (Iron-Deficiency Anemias[Title/Abstract])) OR (Iron Deficiency Anemias[Title/Abstract])) OR (Anemias, Iron-Deficiency[Title/Abstract])) OR (Anemias, Iron Deficiency[Title/Abstract])) OR (Vitamin B 12 Deficiency[MeSH Terms])) OR (Vitamin B 12 Deficiency[Title/Abstract])) OR (Deficiencies, Vitamin B12[Title/Abstract])) OR (Deficiency, Vitamin B 12[Title/Abstract])) OR (Deficiency, Vitamin B12[Title/Abstract])) OR (Vitamin B12 Deficiencies[Title/Abstract])) OR (Vitamin B12 Deficiency[Title/Abstract])) OR (Vitamin A Deficiency[MeSH Terms])) OR (Vitamin A Deficiency[Title/Abstract])) OR (Deficiencies, Vitamin A[Title/Abstract])) OR (Deficiency, Vitamin A[Title/Abstract])) OR (Vitamin A Deficiencies[Title/Abstract])) OR (Zinc Deficiency[Title/Abstract])) OR (Zinc[MeSH Terms])) OR (Zinc[Title/Abstract])) OR (Growth Disorders[MeSH Terms])) OR (Growth Disorders[Title/Abstract])) OR (Disorder, Growth[Title/Abstract])) OR (Growth Disorder[Title/Abstract])) OR (Stunting[Title/Abstract])) OR (Stuntings[Title/Abstract])) OR (Stunted Growth[Title/Abstract])) OR (Growth, Stunted[Title/Abstract])) OR (Iodine[MeSH Terms])) OR (Iodine[Title/Abstract])) OR (Iodine-127[Title/Abstract])) OR (Iodine 127[Title/Abstract])) OR (Iodine Deficiency[Title/Abstract])) OR (Iodine Deficiency, Primary[Title/Abstract])) OR (Malnutrition[MeSH Terms])) OR (Malnutrition[Title/Abstract])) OR (Nutritional Deficiency[Title/Abstract])) OR (Nutritional Deficiencies[Title/Abstract])) OR (Undernutrition[Title/Abstract])) OR (Malnourishment[Title/Abstract])) OR (Malnourishments[Title/Abstract]))) AND ((((Incidence[MeSH Terms]) OR (Incidence[Title/Abstract])) OR (Incidences[Title/Abstract])) OR (Secondary Attack Rate[Title/Abstract])) OR (Attack Rate, Secondary[Title/Abstract])) OR (Rate, Secondary Attack[Title/Abstract])) OR (Secondary Attack Rates[Title/Abstract])) OR (Incidence Proportion[Title/Abstract])) OR (Incidence Proportions[Title/Abstract])) OR (Proportion, Incidence[Title/Abstract])) OR (Attack Rate[Title/Abstract])) OR (Attack Rates[Title/Abstract])) OR (Rate, Attack[Title/Abstract])) OR (Cumulative Incidence[Title/Abstract])) OR (Cumulative Incidences[Title/Abstract])) OR (Incidence, Cumulative[Title/Abstract])) OR (Incidence Rate[Title/Abstract])) OR (Incidence Rates[Title/Abstract])) OR (Rate, Incidence[Title/Abstract])) OR (Person-time Rate[Title/Abstract])) OR (Person time Rate[Title/Abstract])) OR	162

	(Person-time Rates[Title/Abstract])) OR (Rate, Person-time[Title/Abstract])) OR (Cross-Sectional Studies[MeSH Terms])) OR (Cross-Sectional Studies[Title/Abstract])) OR (Cross Sectional Studies[Title/Abstract])) OR (Cross-Sectional Study[Title/Abstract])) OR (Studies, Cross-Sectional[Title/Abstract])) OR (Study, Cross-Sectional[Title/Abstract])) OR (Cross Sectional Analysis[Title/Abstract])) OR (Analyses, Cross Sectional[Title/Abstract])) OR (Cross Sectional Analyses[Title/Abstract])) OR (Disease Frequency Surveys[Title/Abstract])) OR (Cross-Sectional Survey[Title/Abstract])) OR (Cross Sectional Survey[Title/Abstract])) OR (Cross-Sectional Surveys[Title/Abstract])) OR (Survey, Cross-Sectional[Title/Abstract])) OR (Surveys, Cross-Sectional[Title/Abstract])) OR (Surveys, Disease Frequency[Title/Abstract])) OR (Disease Frequency Survey[Title/Abstract])) OR (Survey, Disease Frequency[Title/Abstract])) OR (Analysis, Cross-Sectional[Title/Abstract])) OR (Analyses, Cross-Sectional[Title/Abstract])) OR (Analysis, Cross Sectional[Title/Abstract])) OR (Cross-Sectional Analyses[Title/Abstract])) OR (Cross-Sectional Analysis[Title/Abstract])) OR (Prevalence Studies[Title/Abstract])) OR (Prevalence Study[Title/Abstract])) OR (Studies, Prevalence[Title/Abstract])) OR (Study, Prevalence[Title/Abstract]))	
Lilacs/BVS	(Adult) OR (mh:(Adult)) OR (Adults) OR (Young Adult) OR (Young Adult) OR (Adult, Young) OR (Adults, Young) OR (Young Adults) OR (Middle Aged) OR (middle aged) OR (Middle Age) AND (Food Insecurity) OR (mh:(Food Insecurity)) OR (Food Insecurities) OR (Insecurities, Food) OR (Insecurity, Food) AND (famine) OR (mh:(famine)) OR (Iron) OR (iron) OR (Iron-56) OR (Iron 56) OR (Iron Deficiency) OR (Anemia, Iron-Deficiency) OR (Anemia, Iron-Deficiency) OR (Anemia, Iron Deficiency) OR (Iron-Deficiency Anemia) OR (Iron Deficiency Anemia) OR (Iron-Deficiency Anemias) OR (Iron Deficiency Anemias OR Anemias) OR (Iron-Deficiency) OR (Anemias, Iron Deficiency) OR (Vitamin B 12 Deficiency) OR (Vitamin B 12 Deficiency) OR (Deficiencies, Vitamin B12) OR (Deficiency, Vitamin B 12) OR (Deficiency, Vitamin B12) OR (Vitamin B12 Deficiencies) OR (Vitamin B12 Deficiency) OR (Vitamin A Deficiency) OR (Vitamin A Deficiency) OR (Deficiencies, Vitamin A) OR (Deficiency, Vitamin A) OR (Vitamin A Deficiencies) OR (Zinc Deficiency) OR (Zinc) OR (Growth Disorders) OR (Growth Disorders) OR (Disorder, Growth) OR (Growth Disorder) OR (Stunting) OR (Stuntings) OR (Stunted Growth) OR (Growth, Stunted) OR (Iodine) OR (Iodine) OR (Iodine-127) OR (Iodine 127) OR (Iodine Deficiency) OR (Iodine Deficiency, Primary) OR (Malnutrition) OR (Malnutrition) OR (Nutritional Deficiency) OR (Nutritional Deficiencies) OR (Undernutrition) OR (Malnourishment) OR (Malnourishments) AND (Incidence) OR (mh:(Incidence)) OR (Incidences) OR (Incidences) OR (Secondary Attack Rate) OR (Attack Rate, Secondary) OR (Rate, Secondary Attack) OR (Secondary Attack Rates) OR (Incidence Proportion) OR (Incidence Proportions) OR (Proportion, Incidence) OR (Attack Rate) OR (Attack Rates) OR (Rate, Attack) OR (Cumulative Incidence) OR (Cumulative Incidences) OR (Incidence, Cumulative) OR (Incidence Rate) OR (Incidence Rates) OR (Rate, Incidence) OR (Person-time Rate) OR (Person time Rate) OR (Person-time Rates) OR (Rate, Person-time) OR (Cross-Sectional Studies) OR (Cross-Sectional Studies) OR (Cross Sectional Studies) OR (Cross-Sectional Study) OR (Studies, Cross-Sectional) OR (Study, Cross-Sectional) OR (Cross Sectional Analysis) OR (Analyses, Cross Sectional) OR (Cross Sectional Analyses) OR (Disease Frequency Surveys) OR (Cross-Sectional Survey) OR (Cross Sectional Survey) OR (Cross-Sectional Surveys) OR (Survey, Cross-Sectional) OR (Surveys, Cross-Sectional) OR (Surveys, Disease Frequency) OR (Disease Frequency Survey) OR (Survey,	98

	Disease Frequency) OR (Analysis, Cross-Sectional) OR (Analyses, Cross-Sectional) OR (Analysis, Cross Sectional) OR (Cross-Sectional Analyses) OR (Cross-Sectional Analysis) OR (Prevalence Studies) OR (Prevalence Study) OR (Studies, Prevalence) OR (Study, Prevalence)	
Web of sciense	#1 (((((((TS=(Adult)) OR TS=(Adults)) OR TS=(Young Adult)) OR TS=(Adult, Young)) OR TS=(Adults, Young)) OR TS=(Young Adults)) OR TS=(Middle Aged)) OR TS=(middle aged)) OR TS=(Middle Age)<br><br>#2 ((((TS=(Food Insecurity)) OR TS=(Food Insecurity)) OR TS=(Food Insecurities)) OR TS=(Insecurities, Food)) OR TS=(Insecurity, Food)<br><br>#3 ((TS=(famine)) OR TS=(Iron)) OR TS=(iron)) OR TS=(Iron-56)) OR TS=(Iron 56)) OR TS=(Iron Deficiency)) OR TS=(Anemia, Iron-Deficiency)) OR TS=(Anemia, Iron-Deficiency)) OR TS=(Anemia, Iron Deficiency)) OR TS=(Iron-Deficiency Anemia)) OR TS=(Iron Deficiency Anemia)) OR TS=(Iron-Deficiency Anemias)) OR TS=(Iron Deficiency Anemias)) OR TS=(Anemias)) OR TS=(Iron-Deficiency)) OR TS=(Anemias, Iron Deficiency)) OR TS=(Vitamin B 12 Deficiency)) OR TS=(Vitamin B 12 Deficiency)) OR TS=(Deficiencies, Vitamin B12)) OR TS=(Deficiency, Vitamin B 12)) OR TS=(Deficiency, Vitamin B12)) OR TS=(Vitamin B12 Deficiencies)) OR TS=(Vitamin B12 Deficiency)) OR TS=(Vitamin A Deficiency)) OR TS=(Vitamin A Deficiency)) OR TS=(Deficiencies, Vitamin A)) OR TS=(Deficiency, Vitamin A)) OR TS=(Vitamin A Deficiencies)) OR TS=(Zinc Deficiency))) OR TS=(Zinc)) OR TS=(Growth Disorders)) OR TS=(Growth Disorders)) OR TS=(Disorder, Growth)) OR TS=(Growth Disorder)) OR TS=(Stunting)) OR TS=(Stuntings)) OR TS=(Stunted Growth)) OR TS=(Growth, Stunted)) OR TS=(Iodine)) OR TS=(Iodine)) OR TS=(Iodine-127)) OR TS=(Iodine 127)) OR TS=(Iodine Deficiency)) OR TS=(Iodine Deficiency, Primary)) OR TS=(Malnutrition)) OR TS=(Malnutrition)) OR TS=(Nutritional Deficiency)) OR TS=(Nutritional Deficiencies)) OR TS=(Undernutrition)) OR TS=(Malnourishment)) OR TS=(Malnourishments)<br><br>#4 (((TS=(Incidence)) OR TS=(Incidences)) OR TS=(Secondary Attack Rate)) OR TS=(Attack Rate, Secondary)) OR TS=(Rate, Secondary Attack)) OR TS=(Secondary Attack Rates)) OR TS=(Incidence Proportion)) OR TS=(Incidence Proportions)) OR TS=(Proportion, Incidence)) OR TS=(Attack Rate)) OR TS=(Attack Rates)) OR TS=(Rate, Attack)) OR TS=(Cumulative Incidence)) OR TS=(Cumulative Incidences)) OR TS=(Incidence, Cumulative)) OR TS=(Incidence Rate)) OR TS=(Incidence Rates)) OR TS=(Rate, Incidence)) OR TS=(Person-time Rate)) OR TS=(Person time Rate)) OR TS=(Person-time Rates)) OR TS=(Rate, Person-time)) OR TS=(Cross-Sectional Studies)) OR TS=(Cross-Sectional Studies)) OR TS=(Cross Sectional Studies)) OR TS=(Cross-Sectional Study)) OR TS=(Studies, Cross-Sectional)) OR TS=(Study, Cross-Sectional)) OR TS=(Cross Sectional Analysis)) OR TS=(Analyses, Cross Sectional)) OR TS=(Cross Sectional Analyses)) OR TS=(Disease Frequency Surveys)) OR TS=(Cross-Sectional Survey)) OR TS=(Cross Sectional Survey)) OR TS=(Cross-Sectional Surveys)) OR TS=(Survey, Cross-Sectional)) OR TS=(Surveys, Cross-Sectional)) OR TS=(Surveys, Disease Frequency)) OR TS=(Disease Frequency Survey)) OR TS=(Survey, Disease Frequency)) OR TS=(Analysis, Cross-Sectional)) OR TS=(Analyses, Cross-Sectional))	151

	OR TS=(Analysis, Cross Sectional)) OR TS=(Cross-Sectional Analyses)) OR TS=(Cross-Sectional Analysis)) OR TS=(Prevalence Studies)) OR TS=(Prevalence Study)) OR TS=(Studies, Prevalence)) OR TS=(Study, Prevalence)<br><br>(((#1) AND #2) AND #3) AND #4	
Embase	('adult'/exp OR adult OR 'adults'/exp OR adults OR 'young adult'/exp OR 'young adult' OR (young AND ('adult'/exp OR adult)) OR 'adult, young'/exp OR 'adult, young' OR (('adult,'/exp OR adult,) AND young) OR 'adults, young' OR (('adults,'/exp OR adults,) AND young) OR 'young adults'/exp OR 'young adults' OR (young AND ('adults'/exp OR adults)) OR 'middle aged'/exp OR 'middle aged' OR (middle AND ('aged'/exp OR aged)) OR 'middle age'/exp OR 'middle age' OR (middle AND ('age'/exp OR age))) AND ('food insecurity'/exp OR 'food insecurity' OR (('food'/exp OR food) AND ('insecurity'/exp OR insecurity)) OR 'food insecurities' OR (('food'/exp OR food) AND insecurities) OR 'insecurities, food' OR (insecurities, AND ('food'/exp OR food)) OR 'insecurity, food' OR (insecurity, AND ('food'/exp OR food))) AND ('famine'/exp OR famine OR 'iron'/exp OR iron OR 'iron 56'/exp OR 'iron 56' OR (('iron'/exp OR iron) AND 56) OR 'iron deficiency'/exp OR 'iron deficiency' OR (('iron'/exp OR iron) AND ('deficiency'/exp OR deficiency)) OR 'anemia, iron-deficiency'/exp OR 'anemia, iron-deficiency' OR (('anemia,'/exp OR anemia,) AND ('iron deficiency'/exp OR 'iron deficiency')) OR 'anemia, iron deficiency'/exp OR 'anemia, iron deficiency' OR (('anemia,'/exp OR anemia,) AND ('iron'/exp OR iron) AND ('deficiency'/exp OR deficiency)) OR 'iron-deficiency anemia'/exp OR 'iron-deficiency anemia' OR (('iron deficiency'/exp OR 'iron deficiency') AND ('anemia'/exp OR anemia)) OR 'iron deficiency anemia'/exp OR 'iron deficiency anemia' OR (('iron'/exp OR iron) AND ('deficiency'/exp OR deficiency) AND ('anemia'/exp OR anemia)) OR 'iron-deficiency anemias' OR (('iron deficiency'/exp OR 'iron deficiency') AND anemias) OR 'iron deficiency anemias' OR (('iron'/exp OR iron) AND ('deficiency'/exp OR deficiency) AND anemias) OR 'anemias, iron-deficiency' OR (anemias, AND ('iron deficiency'/exp OR 'iron deficiency')) OR 'anemias, iron deficiency' OR (anemias, AND ('iron'/exp OR iron) AND ('deficiency'/exp OR deficiency)) OR 'vitamin b 12 deficiency'/exp OR 'vitamin b 12 deficiency' OR (('vitamin'/exp OR vitamin) AND b AND 12 AND ('deficiency'/exp OR deficiency)) OR 'deficiencies, vitamin b12' OR (deficiencies, AND ('vitamin'/exp OR vitamin) AND ('b12'/exp OR b12)) OR 'deficiency, vitamin b 12' OR (deficiency, AND ('vitamin'/exp OR vitamin) AND b AND 12) OR 'deficiency, vitamin b12' OR (deficiency, AND ('vitamin'/exp OR vitamin) AND ('b12'/exp OR b12)) OR 'vitamin b12 deficiencies' OR (('vitamin'/exp OR vitamin) AND ('b12'/exp OR b12) AND deficiencies) OR 'vitamin b12 deficiency'/exp OR 'vitamin b12 deficiency' OR (('vitamin'/exp OR vitamin) AND ('b12'/exp OR b12) AND ('deficiency'/exp OR deficiency)) OR 'vitamin a deficiency'/exp OR 'vitamin a deficiency' OR (('vitamin'/exp OR vitamin) AND a AND ('deficiency'/exp OR deficiency)) OR 'deficiencies, vitamin a' OR (deficiencies, AND ('vitamin'/exp OR vitamin) AND a) OR 'deficiency, vitamin a'/exp OR 'deficiency, vitamin a' OR (deficiency, AND ('vitamin'/exp OR vitamin) AND a) OR 'vitamin a deficiencies' OR (('vitamin'/exp OR vitamin) AND a AND deficiencies) OR 'zinc deficiency'/exp OR 'zinc deficiency' OR (('zinc'/exp OR zinc) AND ('deficiency'/exp OR deficiency)) OR 'zinc'/exp OR zinc OR 'growth disorders'/exp OR 'growth disorders' OR (('growth'/exp OR growth) AND ('disorders'/exp OR disorders)) OR 'disorder, growth'/exp OR 'disorder, growth' OR (('disorder,'/exp OR disorder,)	492

	AND ('growth'/exp OR growth)) OR 'growth disorder'/exp OR 'growth disorder' OR (('growth'/exp OR growth) AND ('disorder'/exp OR disorder)) OR 'stunting'/exp OR stunting OR stuntings OR 'stunted growth'/exp OR 'stunted growth' OR (stunted AND ('growth'/exp OR growth)) OR 'growth, stunted' OR (('growth,'/exp OR growth,) AND stunted) OR 'iodine'/exp OR iodine OR 'iodine 127'/exp OR 'iodine 127' OR (('iodine'/exp OR iodine) AND 127) OR 'iodine deficiency'/exp OR 'iodine deficiency' OR (('iodine'/exp OR iodine) AND ('deficiency'/exp OR deficiency)) OR 'iodine deficiency, primary' OR (('iodine'/exp OR iodine) AND deficiency, AND primary) OR 'malnutrition'/exp OR malnutrition OR 'nutritional deficiency'/exp OR 'nutritional deficiency' OR (nutritional AND ('deficiency'/exp OR deficiency)) OR 'nutritional deficiencies' OR (nutritional AND deficiencies) OR 'undernutrition'/exp OR undernutrition OR 'malnourishment'/exp OR malnourishment OR malnourishments) AND ('incidence'/exp OR incidence OR incidences OR 'secondary attack rate'/exp OR 'secondary attack rate' OR (('secondary'/exp OR secondary) AND attack AND rate) OR 'attack rate, secondary' OR (attack AND rate, AND ('secondary'/exp OR secondary)) OR 'rate, secondary attack' OR (rate, AND ('secondary'/exp OR secondary) AND attack) OR 'secondary attack rates' OR (('secondary'/exp OR secondary) AND attack AND rates) OR 'incidence proportion'/exp OR 'incidence proportion' OR (('incidence'/exp OR incidence) AND proportion) OR 'incidence proportions' OR (('incidence'/exp OR incidence) AND proportions) OR 'proportion, incidence' OR (proportion, AND ('incidence'/exp OR incidence)) OR 'attack rate'/exp OR 'attack rate' OR (attack AND rate) OR 'attack rates' OR (attack AND rates) OR 'rate, attack' OR (rate, AND attack) OR 'cumulative incidence'/exp OR 'cumulative incidence' OR (cumulative AND ('incidence'/exp OR incidence)) OR 'cumulative incidences' OR (cumulative AND incidences) OR 'incidence, cumulative' OR (('incidence,'/exp OR incidence,) AND cumulative) OR 'incidence rate'/exp OR 'incidence rate' OR (('incidence'/exp OR incidence) AND rate) OR 'incidence rates' OR (('incidence'/exp OR incidence) AND rates) OR 'rate, incidence'/exp OR 'rate, incidence' OR (rate, AND ('incidence'/exp OR incidence)) OR 'person-time rate'/exp OR 'person-time rate' OR ('person time' AND rate) OR 'person time rate'/exp OR 'person time rate' OR (person AND ('time'/exp OR time) AND rate) OR 'person-time rates' OR ('person time' AND rates) OR 'rate, person-time' OR (rate, AND 'person time') OR 'cross-sectional studies'/exp OR 'cross-sectional studies' OR ('cross sectional' AND ('studies'/exp OR studies)) OR 'cross sectional studies'/exp OR 'cross sectional studies' OR (cross AND sectional AND ('studies'/exp OR studies)) OR 'cross-sectional study'/exp OR 'cross-sectional study' OR ('cross sectional' AND ('study'/exp OR study)) OR 'studies, cross-sectional' OR (studies, AND 'cross sectional') OR 'study, cross-sectional' OR (('study,'/exp OR study,) AND 'cross sectional') OR 'cross sectional analysis'/exp OR 'cross sectional analysis' OR (cross AND sectional AND ('analysis'/exp OR analysis)) OR 'analyses, cross sectional' OR (analyses, AND cross AND sectional) OR 'cross sectional analyses' OR (cross AND sectional AND analyses) OR 'disease frequency surveys' OR (('disease'/exp OR disease) AND ('frequency'/exp OR frequency) AND ('surveys'/exp OR surveys)) OR 'cross-sectional survey' OR ('cross sectional' AND ('survey'/exp OR survey)) OR 'cross sectional survey' OR (cross AND sectional AND ('survey'/exp OR survey)) OR 'cross-sectional surveys' OR ('cross sectional' AND ('surveys'/exp OR surveys)) OR 'survey, cross-sectional' OR (survey, AND 'cross sectional') OR 'surveys, cross-sectional' OR (surveys, AND 'cross sectional') OR 'surveys, disease frequency' OR (surveys, AND ('disease'/exp OR disease) AND ('frequency'/exp OR frequency)) OR 'disease frequency survey' OR (('disease'/exp OR disease) AND ('frequency'/exp OR frequency) AND ('survey'/exp OR survey)) OR 'survey, disease frequency' OR (survey, AND ('disease'/exp OR disease) AND ('frequency'/exp OR frequency)) OR 'analysis, cross-sectional' OR (('analysis,'/exp OR analysis,) AND 'cross sectional') OR 'analyses, cross-sectional' OR (analyses, AND 'cross sectional') OR 'analysis, cross	

	sectional' OR (('analysis,'/exp OR analysis,) AND cross AND sectional) OR 'cross-sectional analyses' OR ('cross sectional' AND analyses) OR 'cross-sectional analysis' OR ('cross sectional' AND ('analysis'/exp OR analysis)) OR 'prevalence studies' OR (('prevalence'/exp OR prevalence) AND ('studies'/exp OR studies)) OR 'prevalence study'/exp OR 'prevalence study' OR (('prevalence'/exp OR prevalence) AND ('study'/exp OR study)) OR 'studies, prevalence' OR (studies, AND ('prevalence'/exp OR prevalence)) OR 'study, prevalence' OR (('study,'/exp OR study,) AND ('prevalence'/exp OR prevalence)))	
CINAHL	(AB famine OR AB famine OR AB Iron OR AB iron OR AB Iron-56 OR AB Iron 56 OR AB Iron Deficiency OR AB Anemia, Iron-Deficiency OR AB Anemia, Iron-Deficiency OR AB Anemia, Iron Deficiency OR AB Iron-Deficiency Anemia OR AB Iron Deficiency Anemia OR AB Iron-Deficiency Anemias OR AB Iron Deficiency Anemias OR AB Anemias, Iron-Deficiency OR AB Anemias, Iron Deficiency OR AB Vitamin B 12 Deficiency OR AB Vitamin B 12 Deficiency OR AB Deficiencies, Vitamin B12 OR AB Deficiency, Vitamin B 12 OR AB Deficiency, Vitamin B12 OR AB Vitamin B12 Deficiencies OR AB Vitamin B12 Deficiency OR AB Vitamin A Deficiency OR AB Vitamin A Deficiency OR AB Deficiencies, Vitamin A OR AB Deficiency, Vitamin A OR AB Vitamin A Deficiencies OR AB Zinc Deficiency OR AB Zinc OR AB Zinc OR AB Growth Disorders OR AB Growth Disorders OR AB Disorder, Growth OR AB Growth Disorder OR AB Stunting OR AB Stuntings OR AB Stunted Growth OR AB Growth, Stunted OR AB Iodine OR AB Iodine OR AB Iodine-127 OR AB Iodine 127 OR AB Iodine Deficiency OR AB Iodine Deficiency, Primary OR AB Malnutrition OR AB Malnutrition OR AB Nutritional Deficiency OR AB Nutritional Deficiencies OR AB Undernutrition OR AB Malnourishment OR AB Malnourishments) AND (S1 AND S2 AND S3 AND S4)	21

Suplementary Material File S3. Risk of bias assessment

Table. Risk of bias for each individual study assessed by Joanna Briggs Institute critical appraisal checklist for prevalence studies

**Studies**	**Criteria**							
	**1***	**2***	**3***	**4***	**5***	**6***	**7***	**8***
Dixon et al. (2001)	Y	Y	Y	Y	Y	Y	Y	Y
Egeland et al (2011)	Y	Y	Y	Y	Y	Y	Y	Y
Gowda, Hadley, Aiello (2012)	N	Y	Y	Y	Y	Y	Y	Y
Jamieson et al (2012)	Y	Y	Y	Y	Y	Y	Y	Y
Fischer et al. (2014)	Y	Y	Y	Y	Y	Y	Y	Y
McDonald et al (2015)	Y	Y	Y	Y	Y	Y	Y	Y
Sekhar et al (2016)	Y	Y	Y	Y	Y	Y	Y	Y
Ghose et al. (2016)	N	N	Y	Y	Y	Y	Y	Y
Weigel et al. (2016)	Y	Y	Y	Y	Y	Y	Y	Y
Parker et al (2017)	Y	Y	Y	Y	Y	Y	Y	Y
Soofi et al (2017)	Y	Y	Y	Y	N	N	Y	Y
Jones et al (2017)	Y	Y	Y	Y	Y	Y	Y	Y
Habib et al (2018)	Y	Y	Y	Y	Y	Y	Y	Y
Mastiholi et al. (2018)	Y	N	Y	Y	N	N	Y	Y
Murillo-Castillo et al (2018)	Y	N	Y	Y	Y	Y	Y	Y
Kazemi et al (2020)	Y	Y	Y	Y	Y	Y	Y	Y
Pobee et al (2020)	Y	Y	Y	Y	Y	Y	Y	Y
Lopes et al (2022) [1]	Y	Y	Y	Y	Y	Y	Y	Y

Y = Yes, N = No, U = Unclear, NA = Not applicable

1* Criteria for inclusion in the sample clearly defined;

2* Study subjects and the setting described in detail;

3* Exposure measured in a valid and reliable way;

4* Objective and standard criteria for measurement;

5* Confounding factors identified;

6* Strategies to deal with confounding factors;

7* Outcomes measured in a valid and reliable way;

8* Appropriate statistical analysis.

[1] Researcher SOL did not participate in the quality assessment of this article.
